# Building community resilience to climate change: The role of a Population-Health-Environment programme in supporting the community response to cyclone Haruna in Madagascar

**DOI:** 10.4102/jamba.v12i1.730

**Published:** 2020-01-27

**Authors:** Vikas Mohan, Karen Hardee, Caroline Savitzky

**Affiliations:** 1 Blue Ventures Conservation, London, United Kingdom; 2 What Works Association, Morristown, New Jersey, United States; 3 Boston Medical Center, Boston, United States

## Introduction

Tropical cyclone Haruna made landfall in southwest Madagascar in February 2013; it was a powerful Category 2 storm with heavy rain and wind speeds of 150 km/h, making it the largest cyclone that this region had experienced in more than 35 years. Houses, schools, government buildings and health clinics throughout the communes of Befandefa and Morombe (home to approximately 15 000 people) were damaged, with many completely destroyed.

Communities in this area of southwest Madagascar rely almost exclusively on fishing for food and livelihood. The heavy wind and rain meant that families were unable to fish for 1–2 weeks, resulting in acute food shortages for many. Flooding and contamination of water sources triggered outbreaks of diarrhoea and malaria, whilst also preventing aid from reaching the affected area. This area was inaccessible by road for over 6 weeks, leaving travel by boat, ox cart or on foot (sometimes wading through water that was chest-deep) as the only ways to reach the area.

Yet, in the days and weeks after the cyclone, the coastal communities affected by this storm coordinated and executed a rapid response to the disaster, ensuring that important information was collected and disseminated and that people’s immediate needs for shelter, food and medical treatment were met. This article examines the community’s response, and how this response was strengthened by a community-level Population, Health and Environment (PHE) programme in these regions. This is followed by the authors’ views on how this approach can contribute to improved community resilience to climatic shocks and other extreme weather events, and more broadly, how this approach might support communities to become more resilient to climate change.

## Defining community resilience

Amongst the many overlapping definitions of community resilience is the following: The collective ability of a neighbourhood or geographically defined area to deal with stressors and efficiently resume the rhythms of daily life through cooperation following shocks (Aldrich & Meyer 2015).

The most important force tying communities together and boosting community resilience is social capital, or ‘the aggregate of resources that arise from reciprocal social relationships in formal and informal networks, fuelling community’s ability to achieve desired results through collective action’ (Mancini & Bowen 2009:255). Central to the idea of social capital is the importance of social networks: networks between emotionally close community members, such as friends and relatives; networks created through community activities and organisations; networks between regular citizens and those in power (Aldrich & Meyer 2015).

## Blue Ventures’ population-health-environment programme in southwest Madagascar

Blue Ventures (BV) is an international marine conservation non-government organisation that works with coastal communities to help rebuild tropical fisheries and catalyse community-led marine conservation. It has been working with fishing communities in southwest Madagascar since 2003. Recognising the interconnections between human and environmental health, their approach to working with coastal communities includes supporting locally led management of fisheries and marine ecosystems, supporting the development of alternative livelihoods (farming of seaweed and sea cucumber) and providing community-based health services. Periodic temporary fisheries closures support the recovery of economically important fisheries, with a corresponding boost in income from fishing. Communities from across 26 villages work collectively to manage Madagascar’s first locally managed marine area (LMMA), an area of 500 km^2^ of nearshore waters, where destructive fishing practices are prohibited and vital marine habitats are protected. The LMMA is governed by a management committee, comprising elected members from the local community. To address unmet health needs, local women have been trained as community health workers (CHWs) to provide health care and education to their communities, focussing on family planning, maternal and child health care and safe water initiatives. This community health care provision and education (which has been supported by outreach services from Marie Stopes Madagascar and other health partners) have been fully integrated into BV’s programme of activities, with a single overarching vision for enabling communities to live and manage their marine resources sustainably.

This multisector approach, often referred to as PHE because it recognises the interconnections between human populations, their health and their environment, has led to dramatic improvements in health (Robson et al. 2017) and in income from fishing (Oliver et al. 2015).

## Cyclone Haruna: Community response

As the winds and rain from the cyclone ceased, the importance of social capital became immediately apparent. Social systems, trust-based networks between friends, families, community organisation members, citizens and institutions were critical in carrying out relief measures following Haruna, and no time was lost in determining who should lead or how tasks were to be achieved. Families supported each other, often working with their extended family and neighbours to rebuild each other’s homes. (Repairing homes was one of the most easily implementable actions as materials could be gathered locally.) Through informal social networks, community leaders were quickly made aware of the families that had suffered the most damage and who was in the most need.

These informal social networks were also seen linking with formal institutions in order to ensure that assistance reached where it was needed. The CHWs, village leaders and the management committee of the LMMA immediately adopted leadership functions. They gathered information, coordinated activities with government authorities and most notably, convened water committees to treat well water in order to make it safe for drinking. They also stood guard over the public wells throughout the night to ensure treatments were completed and that they were not overused.

Community health workers, as trusted community members, quickly began holding community education meetings on post-cyclone health risks. They increased the distribution of supplies that they were already trained to provide, such as mosquito nets for people sleeping outside whilst rebuilding their homes, and water treatment products and oral rehydration solution for children with diarrhoea. Blue Ventures simultaneously worked with community leaders, CHWs and others to manage supply chains and transportation, sharing information and coordinating with national disaster response teams. As a network of women embedded within the communities the CHWs served, they were particularly well-placed to provide accurate and up-to-date information to community leaders and disaster response teams.

Community health workers and community members also travelled for hours in ox carts almost completely submerged in water to ensure that the outreach family planning clinic schedule could be maintained. They carried medical supplies to areas that were completely inaccessible to national disaster response teams. Their work and dedication to their community ensured that regularly scheduled family planning clinics continued to take place in spite of the disruption caused by the cyclone. Many people lost their health cards along with their other belongings during the cyclone. As they were receiving services through the CHWs, who had records of their appointments and follow-up details, they were not at risk of being lost to care.

Maintenance of routine health care services, coupled with the provision of additional services and health education where needed, enabled those in need to access food, shelter and support for rebuilding their homes, and served to limit the impact of the cyclone. Communities could thus resume normal functioning more quickly. In spite of the devastating impacts of Haruna, the authors believe that by working cooperatively to respond to this extreme weather event and in resuming their normal activities as quickly as possible, these communities were clearly exhibiting community resilience.

## Discussion

If, as we believe, these communities demonstrated a degree of resilience, how did BV’s PHE programme contribute to this? Were there gaps in the communities’ response that could be used to help inform programme development or improve responses to future extreme weather events?

The dramatic improvements in health noted earlier (Robson et al. 2017) are likely to have contributed to community resilience. A community mostly comprising healthy individuals is more resilient, with less healthy individuals able to rely on healthier individuals for physical and emotional support (US Department of Health and Human Services 2009). Improved access to family planning services in particular has been posited to contribute to greater resilience to climate change (Grace 2017; Jiang & Hardee 2011; Kock & Prost 2017; PRB and Worldwatch 2014). Community health workers are also central to maintaining the health of remote/underserved communities in much of the developing world. Their role is to address unmet health care needs, serve as sources of information and provide referrals to health facilities. The rapid response of the network of CHWs in the aftermath of the cyclone contributed tangibly to the community’s recovery. Health service provision was maintained, a health care response aimed at harm minimisation was mounted and as members of their communities, CHWs’ knowledge of their patients ensured that care was not interrupted.

Similarly, a mature and well-functioning leadership committee responsible for governing the locally managed marine area meant that a well-established system was in place to enable information to be shared, decisions to be made and resources to be mobilised. The actions of both of these community groups were central to the community’s response to the cyclone.

An important part of being able to adapt to extreme weather events is the diversification of livelihoods to include less weather-dependent income-generating activities. Whilst community-based aquaculture was well established, these activities were not developed with climate resilience in mind and were disrupted by the cyclone. Sea cucumber pens were damaged and seaweed lines were lost. It would also not have been possible in the short term to increase income from aquaculture to cover the immediate shortfall in income from fishing. This meant that even with greater income from fisheries (Oliver et al. 2015), those most affected may not have had access to the financial resources needed to rebuild their homes or buy food. Diversification of livelihoods to include activities that have the potential to yield greater income when needed might enable these communities to recover from such an event more quickly and be better prepared for potential future events. Better access to credit or savings opportunities could similarly enable families to access the money they need to buy food and rebuild their homes. Partly in recognition of this need, community savings groups are now being developed in this region.

Also notable for their absence were well-functioning early warning systems, or any locally developed emergency and disaster response plans. Discussions with community members and leaders in the years after the cyclone have highlighted actions that individuals or families are taking to prepare for future storms, but there is little evidence of planning at the community level. Better access to meteorological information by community members is now possible, thanks to better Internet access and a more widespread use of smartphones. Developing a community-led early warning system should therefore be achievable. Similarly, the community organisation capacity evident in the aftermath of the cyclone suggests that such a system could be linked to a disaster response plan, providing an opportunity for strengthening community resilience for future extreme weather events.

## Population-Health-Environment programme as a model for climate-resilient development

A heavey reliance on fisheries, rapidly growing coastal populations with poor access to health services and limited opportunity to prepare for extreme weather events are challenges faced by so many of the world’s tropical coastal communities beyond the southwest of Madagascar. The PHE programmes, with their emphasis on multisector, community-driven approaches to improve and maintain human and ecosystem well-being, often address many of the root causes of vulnerability to climate change. These programmes typically address unmet health needs (including family planning) alongside initiatives to support sustainable natural resource management and community engagement in biodiversity conservation.

However, there is little documented evidence on whether or not these programmes support communities to become more resilient to climate change, or to recover from extreme weather events (Yavinsky et al. 2015). Responding to this lack of evidence, the Evidence Project (Population Council and Population Reference Bureau) collected and analysed data at a PHE programme site in Tanzania, called Tuungane, to measure resilience and its association with family planning, and to determine the contribution of this PHE programme to building resilience (Hardee et al. 2018). Results of this analysis show that resilience can be measured in the context of PHE programme and that there is a positive link between family planning and resilience. These findings, along with the experiences outlined in this article, could help inform programme design for those working to build community resilience to face climatic changes.
